# 

**Published:** 1809-09

**Authors:** 


### CORRESPONDENCE.

An "Old Correspondent" complains much of the difficulty of understanding the names of some diseases often mentioned in periodical and other works. He instances *Gastrodynia*, a word he complains not to be met with in his Greek Dictionary, nor, though he conceives a visceral disease, in either of the late learned works on the complaints of the peritoneal cavity.—We sincerely lament with him, that an art, of all others the most difficult and the most important, should be rendered more difficult by an unimportant and useless addition to its terms; and we allow with him that it might be as easy to write *pain in the stomach* in English, as to have recourse to a Greek combination. Indeed, the English expression appears to us preferable, in as much as it is more precise.——If our Correspondent is not amusing himself and us with his Queries, we recommend him on these occasions to refer to the two volumes of Cullen's Nosology instead of his Greek Dictionary.

We must be better acquainted with Mr. Thompson's sources of information, before we insert his sceptical remarks on the accounts we receive from eye witnesses.

Dr. Walker's and Leodensis' Communications will appear in our next.

A Paper from Mr. Davies; another from an unknown Correspondent, signed Observator; and a Dissertation on the medicinal effects of the Galvanic influence, have been received.

